# The Effect of SBA-15 Surface Modification on the Process of 18β-Glycyrrhetinic Acid Adsorption: Modeling of Experimental Adsorption Isotherm Data

**DOI:** 10.3390/ma12223671

**Published:** 2019-11-07

**Authors:** Michał Moritz, Małgorzata Geszke-Moritz

**Affiliations:** 1 Institute of Chemistry and Technical Electrochemistry, Faculty of Chemical Technology, Poznan University of Technology, Berdychowo 4, 60-965 Poznań, Poland; 2 Department of Pharmaceutical Chemistry, Faculty of Pharmacy, Poznan University of Medical Sciences, Grunwaldzka 6, 60-780 Poznań, Poland

**Keywords:** bioactive agent, silica, mesoporous materials, surface modification, adsorption modeling

## Abstract

This study aimed at the adsorption of 18β-glycyrrhetinic acid (18β-GA), a pentacyclic triterpenoid derivative of oleanane type, onto functionalized mesoporous SBA-15 silica and non-porous silica (Aerosil^®^) as the reference adsorbent. Although 18β-GA possesses various beneficial pharmacological properties including antitumor, anti-inflammatory, and antioxidant activity, it occurs is small amounts in plant materials. Thus, the efficient methods of this bioactive compound enrichment from vegetable raw materials are currently studied. Siliceous adsorbents were functionalized while using various alkoxysilane derivatives, such as (3-aminopropyl)trimethoxysilane (APTMS), [3-(methylamino)propyl]trimethoxysilane (MAPTMS), (N,N-dimethylaminopropyl)trimethoxysilane (DMAPTMS), and [3-(2-aminothylamino)propyl] trimethoxysilane (AEAPTMS). The effect of silica surface modification with agents differing in the structure and the order of amine groups on the adsorption capacity of the adsorbent and adsorption efficiency were thoroughly examined. The equilibrium adsorption data were analyzed while using the Langmuir, Freundlich, Redlich-Peterson, Temkin, Dubinin-Radushkevich, and Dubinin-Astakhov isotherms. Both linear regression and nonlinear fitting analysis were employed in order to find the best-fitted model. The adsorption isotherms of 18β-GA onto silicas functionalized with APTMS, MAPTMS, and AEAPTMS indicate the Langmuir-type adsorption, whereas sorbents modified with DMAPTMS show the constant distribution of the adsorbate between the adsorbent and the solution regardless of silica type. The Dubinin-Astakhov, Dubinin-Radushkevich, and Redlich-Peterson equations described the best the process of 18β-GA adsorption onto SBA-15 and Aerosil^®^ silicas that were functionalized with APTMS, MAPTMS, and AEAPTMS, regardless of the method that was used for the estimation of isotherm parameters. Based on nonlinear fitting analysis (Dubinin-Astakhov model), it can be concluded that SBA-15 sorbent that was modified with APTMS, MAPTMS, and AEAPTMS is characterized by twice the adsorption capacity (202.8–237.3 mg/g) as compared to functionalized non-porous silica (118.2–144.2 mg/g).

## 1. Introduction

Mesoporous materials are characterized by the pore size from 2 to 50 nm, according to the International Union of Pure and Applied Chemistry (IUPAC) nomenclature [1].

The discovery of a family of ordered mesoporous silica molecular sieves, known as M41S by the researchers from Mobil Oil Company in 1992 [2], started the new era in application of siliceous materials in various fields of science. It is worth mentioning that, in 1971, Chiola et al. [3] first reported on the formation of low-bulk density silica. However, because of the fact of limited characteristics of this material, it has not gained much interest in widespread use. In the last three decades, numerous scientific groups have been working on the development of synthesis methods, leading to the fabrication of new mesoporous structures. It can be observed that the trend in mesoporous material synthesis has been directed from inorganic materials, including silica through hybrid structures, such as metal organic frameworks (MOFs) [4] and periodic mesoporous organosilica (PMO) [5], towards pure organic materials, including porous organic frameworks (POFs) [6]. 

Mesoporous molecular sieves are extremely attractive materials that have found applications in many fields of science e.g., catalysis [7]. They have proved their utilities as heterogeneous catalysts [8], encapsulated catalysts [9], and in photocatalytic hydrogen production [10]. Furthermore, mesoporous matrices can be used as unique supports for immobilization of various catalysts [11,12,13] and have the benefits of simple recovery and reuse after the accomplishment of reactions [14]. These materials have also found interesting applications in the field of electrochemistry [15], solar cells [16], pollutant adsorption [17], and battery components [18]. Mesoporous molecular sieves have unique characteristics, such as high surface area, tunable pore size, large pore volumes, uniform porosity, high mechanical strength, good thermal stability, and, in some cases, excellent biocompatibility [19,20]. Furthermore, mesoporous materials ensure the facile functionalization with different organic groups [21]. A large surface area of mesoporous supports might result in drug release enhancement by molecular dispersion [22], meanwhile the possibility of surface modification might provide the enhancement of adsorption capacity [23] and selective substance binding [24]. The feature of pore diameter tunability may be useful in optimized drug release [25]. The biocompatibility of chosen mesoporous materials determines their usage in tissue regeneration [26], whereas electric conduction is exploited in the application of mesoporous carbon as working electrode component [27].

Depending on their chemical nature, these structures may exhibit magnetic [28], conducting [29], or fluorescent [30] properties. All of these features make mesoporous substances very attractive tools to be used in biomedical applications, including drug delivery [31], biosensing [32], cell imaging [33], protein isolation [34], and many others [21]. 

SBA-15 belongs to the family of ordered mesoporous silicas [35]. It consists of parallel cylindrical pores with hexagonal arrangement. It is characterized by large surface area (up to 800 m^2^/g), high pore volumes, and remarkable hydrothermal stability [35]. Unique properties of mesoporous silicas make them promising matrices for the adsorption of numerous substances, including drugs [36,37,38,39,40], antibacterial agents [41], proteins [42,43,44], and nucleic acids [45]. Mesoporous siliceous materials were also applied as the sorbents in simple, rapid, and reproducible procedures for quantitative analysis of various biologically active molecules. Mirabi et al. [46] used the nanocomposite consisting of SBA-15 silica and graphene oxide for separation, preconcentration, and determination of trace amounts of rutoside in the samples of blood plasma and urine. Amine-functionalized SBA-15 material was applied for the removal and recovery of hydroxytyrosol and tyrosol in olive mill wastewater [47]. The application of SBA-15 and MCF silicas that were modified with amine functions for the adsorption of caffeic acid was reported [48]. Pure and propyl-sulfonic acid-modified SBA-15, SBA-16, MCF, and PHTS materials were employed as efficient sorbents for boldine alkaloid [49,50,51]. Kohno and co-workers [52] used HMS type mesoporous silica functionalized with *n*-propyl groups and containing small amount of aluminum for the successful adsorption of natural anthocyanin dye. Improved stability of adsorbed anthocyanin against visible irradiation was achieved by the utilization of HMS material containing Fe^3+^ [53]. The other group used SBA-15 silica for the preconcentration of quercetin, resveratrol, catechin, epicatechin, rutin, vanillic acid, caffeic acid, and syringic acid [54]. Listed bioactive polyphenols were adsorbed from a Cabernet Sauvignon bottled wine. The results of the performed studies revealed that SBA-15 is an excellent adsorbent for polyphenols from red wine and it can be considered as an alternative material for the extraction of quercetin.

**Figure 1:**
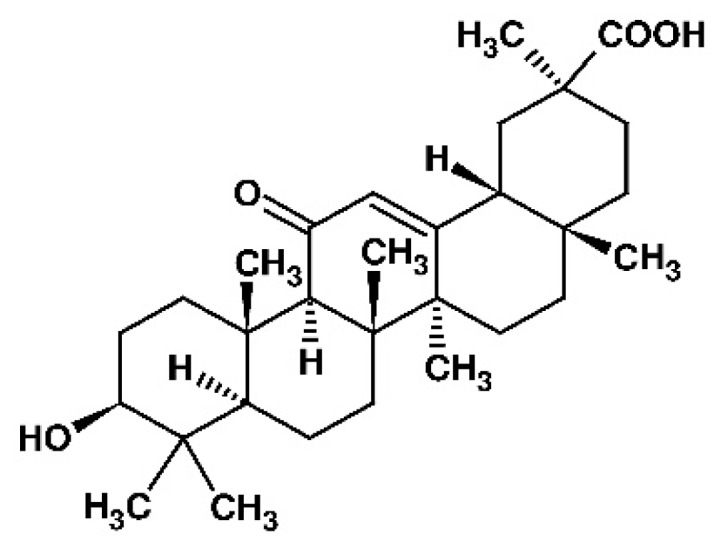
Chemical structure of 18β-glycyrrhetinic acid (18β-GA).

18β-Glycyrrhetinic acid (18β-GA) is a pentacyclic triterpenoid derivative of oleanane type (β-amyrin) that is found in the roots and rhizomes of licorice (*Glycyrrhiza glabra*) [55,56,57]. 18β-GA is an aglycone and active metabolite of glycyrrhizin [55,58]. Figure 1 prevents the chemical structure of 18β-GA. 

18β-GA possesses various beneficial pharmacological properties, including antitumor [55,56], anti-inflammatory [59,60,61], antioxidant [57,58,61], immunomodulatory [56], antiviral [57,58,62], hepatoprotective [57,62,63], antiulcer [56,57,62], and antiallergic [61] activities. Its chemopreventive effect is ascribed to the inhibition of tumorigenesis and the induction of apoptosis in cancer cells [55]. It is often applied as a targeting ligand of various nanovehicles for the chemotherapy of hepatocytes due to its targeting properties [64,65,66]. 18β-GA was used to treat various tissue inflammations. As an example, it was shown to attenuate the ultraviolet-induced skin photoaging in a mouse model, mainly by virtue of its antioxidative and anti-inflammatory properties [61]. 18β-GA has been demonstrated to protect against a number of hepatotoxicants, such as carbon tetrachloride, due to its ability to block the bioactivation of this harmful compound by inhibiting cytochrome P450 2E1 activity and its expression [57]. Its protective effect against methotrexate hepatotoxicity through the down-regulation of peroxisome proliferator activated receptor gamma and nuclear factor (erythroid-derived 2)-like 2 was also reported [67]. Very recently, Zhang et al. described the protective effect of 18β-GA against monocrotaline-induced pulmonary arterial hypertension in rats associated to the inhibition of oxidative stress [68]. 18β-GA plays the role of effective natural adjuvant in chemotherapy, attenuating nephrotoxicity of cisplatin, which is the main side effect of this antineoplastic drug [69,70]. Moreover, this triterpenoid derivative was demonstrated to inhibit airway and lung inflammation [59,60]. 18β-GA was also shown to act against cyclophosphamide-induced cystitis through inhibiting inflammatory stress [71].

18β-GA also exhibits other interesting features. As an example, it enhances the activity of chosen antibiotics, such as aminoglycosides and polymyxin B against certain strains of methicillin-resistant *Staphylococcus aureus* [72]. 18β-GA reveals the antileishmanial effect by great reducing the parasite load in experimental visceral leishmaniasis, mainly through nitric oxide upregulation and proinflammatory cytokine expression [56]. Moreover, it was demonstrated to suppress prolactin hyperactivity and reduce antipsychotic-induced hyperprolactinemia [58]. 18β-GA also reveals an antihyperglycemic effect on streptozocin-diabetic rats, which was evidenced by lowered plasma glucose with a simultaneous increase in the insulin secretion [62]. Its beneficial effect on lipolysis and fat deposition in fish was also proved [73]. 

The enrichment of biologically active compounds is of great importance in acquiring valuable plant components from herbal raw materials and for their further analysis while using appropriate analytical technique. The preconcentration of plant active ingredients is a crucial and indispensable part of the whole analytical procedure [74]. Nevertheless, the sample treatments are usually multistep procedures with the subsequent removal of impurities prior to instrumental analysis. Establishing a simple, rapid, and eco-friendly preconcentration approach for the determination of target analytes in plant materials is quite meaningful [74]. Although its beneficial pharmacological effects, 18β-GA occurs in the roots of the *glycyrrhiza* plant species in small amounts. To the best of our knowledge, to the present day, there is no study devoted to the extraction of 18β-GA from herbal raw materials. Several scientific groups implemented improvements of extraction processes of glycyrrhizic acid from licorice. These efforts included the optimization of solvent to solute ratio, determination of the optimal extraction time, setting the right temperature, and the use of microwave or ultrasounds, which resulted in a continuous raise of extraction yield [75,76,77,78]. Due to its limited natural availability and diverse medical and cosmetic applications [79], it seems to be purposeful to search for adsorbents that provide enrichment of 18β-GA from plants extracts. To our best knowledge, the use of mesoporous silica as the adsorbent for this bioactive compound has not yet been described in the literature. Lu et al. adsorbed the derivative of 18β-GA, 3β-D-monoglucuronyl-18β-glycyrrhetinic acid, while using the macroporous resins to separate it from glycyrrhizin hydrolysate [80].

Keeping in mind the attractive physicochemical properties of mesoporous siliceous materials, the aim of this work is to select an efficient adsorbent for the preconcentration of 18β-GA. The SBA-15 mesoporous molecular sieve will be functionalized with four different modifying agents, such as (3-aminopropyl)trimethoxysilane (APTMS), [3-(methylamino)propyl]trimethoxysilane (MAPTMS), (*N,N*-dimethylaminopropyl)trimethoxysilane (DMAPTMS), and [3-(2-aminoethylamino)propyl]trimethoxysilane (AEAPTMS). Non-porous commercial silica (Aerosil^®^) that was functionalized while using the same modifying agents will be used as a reference sample in all adsorption experiments. As can be seen, employed modifying agents are various amine derivatives of trimethoxysilane. These agents differ in the structure and order of amine group. In this work, the role of siliceous structure and surface functionalization in the process of 18β-GA adsorption will be studied. The modeling of the adsorption process will be provided to better understand the mechanisms of adsorbent-adsorbate interactions. Chosen well-known adsorption isotherm models, such as Langmuir, Freundlich, Redlich-Peterson, Temkin, Dubinin-Radushkevich, and Dubinin-Astakhov, will be used. The sets of the adsorption isotherm parameters will be determined while using both linear regression and nonlinear fitting analysis. The Marquardt’s percent standard deviations (MPSD) error function will be applied to find out the most suitable parameters of nonlinear isotherm equations. 

## 2. Materials and Methods 

### 2.1. Chemicals and Materials

18β-Glycyrrhetinic acid (97%), (3-aminopropyl)trimethoxysilane (97%), [3-(methylamino)propyl]trimethoxysilane (97%), (*N,N*-dimethylaminopropyl)trimethoxysilane (96%), [3-(2-aminoethylamino)propyl]trimethoxysilane (97%), tetraethyl orthosilicate (TEOS) (≥ 99.0%), Pluronic^®^ P-123, and hydrochloric acid (purum p.a. ≥ 32.0%) were purchased from Sigma-Aldrich (Poznań, Poland). Aerosil^®^ was supplied from Roth. Chloroform (p.a. ≥ 98.5%), 2-propanol (p.a. ≥ 99.7%), and anhydrous toluene (99.8%) were purchased from Avantor Performance Materials (Gliwice, Poland).

### 2.2. Synthesis of SBA-15 Silica

SBA-15 material was obtained by the hydrothermal method similar to the procedure described by Zhao et al. [35]. The silica was synthesized by dissolving 48.0 g of poly(ethylene glycol) and poly(propylene glycol) block copolymer (Pluronic^®^ P123) in 1800 cm^3^ of aqueous HCl (1.6 mol/dm^3^) at 35 °C. After adding 102.0 g of tetraethylorthosilicate (TEOS), the mixture was stirred at 35 °C for 20 h. The reaction mixture was aged at 100 °C for 24 h, after which the suspension was filtered and washed with distilled water. The product was dried in air and then calcined at 500 °C for 6 h (heating rate 1 °C/min.).

### 2.3. Modification of Siliceous Adsorbents

Organic moieties were introduced onto silica surface by a grafting strategy while using (3-aminopropyl)trimethoxysilane (APTMS), [3-(methylamino)propyl]trimethoxysilane (MAPTMS), (*N,N*-dimethylaminopropyl)trimethoxysilane (DMAPTMS) and [3-(2-aminoethylamino)propyl]trimethoxysilane (AEAPTMS). Typically, 3.0 g of silica powder (SBA-15 or Aerosil^®^) was redried at 110 °C for 24 h and then dispersed in 50 cm^3^ of water-free toluene containing suitable derivative of trimethoxysilane (0.15 mol/dm^3^). Subsequently, the samples were thoroughly mixed. The reaction was performed at 100 °C for 24 h in a borosilicate bottle that was closed with a screw cap with polytetrafluoroethylene (PTFE) membrane gasket. The crude product was then filtered, washed with several volumes of toluene (5 × 50 cm^3^), followed by chloroform (5 × 50 cm^3^). Afterwards, the precipitate was dried at 40 °C for 1 h and the residues of organic solvents were evacuated at 80 °C for 21 h. The APTMS, MAPTMS, DMAPTMS, and AEAPTMS-functionalized silicas were denoted as SBA-15-AP and Aer-AP, SBA-15-MAP and Aer-MAP, SBA-15-DMAP and Aer-DMAP, and SBA-15-AEAP and Aer-AEAP, respectively.

### 2.4. Adsorption Studies

The adsorption studies of 18β-GA onto functionalized silicas were performed in 2-propanol. The initial adsorbate concentrations were in the range from 120 to 6900 mg/dm^3^. The adsorption experiments were realized in vials by adding 0.010 dm^3^ of 18β-GA solution in organic solvent to 0.100 g of adsorbent. The process of adsorption was conducted at 25 °C for 24 h under stirring. The amount of adsorbed 18β-GA in the equilibrium state was determined from the concentrations of triterpenoid derivative in solution before and after the adsorption process, according to the expression (1), meanwhile the percentage of adsorption efficiency E_ads_ (%) was calculated while using Equation (2):

(1) $$Q_{e}=\frac{(C_{0}-C_{e})\cdot V}{m}$$

(2) $$E_{ads}=\left(\frac{C_{0}-C_{e}}{C_{0}}\right)\cdot 100\%$$

where *Q_e_* (mg/g) is the adsorbed amount of triterpenoid derivative in the equilibrium state, *C*_0_ (mg/dm^3^) and *C_e_* (mg/dm^3^) represent the initial and equilibrium 18β-GA concentration, *V* (dm^3^) is the volume of adsorbate solution, and *m* (g) is the mass of silica used in the experiment.

The adsorption equilibrium of 18β-GA was spectrophotometrically determined at the analytical wavelength of 250 nm. Prior to the measurement, the suspension was centrifuged at 3460× *g* for 15 min. and the supernatant was diluted with an appropriate volume of 2-propanol.

### 2.5. Adsorption Modeling

The linear regression and nonlinear fitting analysis were used to analyze the 18β-GA adsorption process onto siliceous sorbents. The equilibrium adsorption data were analyzed while using several well-known isotherm models [81,82,83,84,85], such as Langmuir, Freundlich, Redlich-Peterson, Temkin, Dubinin-Radushkevich, and Dubinin-Astakhov.

The Langmuir model describes the adsorption on the monolayer surface sites [86]. It refers to the adsorption in which each molecule possesses constant enthalpies and sorption activation energy [81]. The Freundlich isotherm describes the non-ideal and reversible sorption taking place on the heterogeneous surface as well as the multilayer adsorption [81]. The presented two adsorption models provide limited insight with regard to the nature and mechanism of adsorption [82]. Especially, the Freundlich isotherm has been recently criticized for its limitation of lacking a fundamental thermodynamic basis and not approaching the Henry’s law [81]. Thus, to describe the adsorption of 18β-GA the Redlich-Peterson, Temkin, Dubinin-Radushkevich, and Dubinin-Astakhov models were alternatively used. Redlich-Peterson is a three-parameter model featuring both the Langmuir and Freundlich isotherm [81]. The isotherm has a linear dependence on concentration in the nominator and an exponential function in the denominator [87]. It can be employed in homogeneous and heterogeneous systems [81]. The Temkin isotherm describes the effects of indirect adsorption interactions [88]. This model assumes that the heat of adsorption of all molecules in the layer would linearly decrease, rather than logarithmic with the coverage [81]. Additionally, the adsorption is characterized by a uniform distribution of binding energy up to its some maximum value [81,88]. The Dubinin-Radushkevich and Dubinin-Astakhov isotherms are based on the adsorption potential theory that was described by Polanyi. These models assume that the adsorption process is related to the micropore volume filling oppositely to layer-by-layer adsorption on the pore walls [82]. The Dubinin-Radushkevich and Dubinin-Astakhov equations include the additional heterogeneity parameter n, which for Dubinin-Radushkevich equation is 2 [48]. Thus, the Dubinin-Astakhov equation in which the heterogeneity factor is an adjustable, experimentally-derived parameter, is more general [82].

Table 1 summarizes the nonlinear Equations (3)–(8) and linear Equations (9)–(14) forms of the employed equations. 

The Dubinin-Radushkevich and Dubinin-Astakhov models are based on the Polanyi adsorption potential *ε* that can be expressed as [82]:

(15) $$\varepsilon =RT\ln\left(\frac{C_{s}}{C_{e}}\right)$$

where *C_s_* (mg/dm^3^) is the 18β-GA solubility and *C_e_* (mg/dm^3^) is an equilibrium concentration of this triterpenoid derivative.

The isotherm parameters were established while using linear regression and nonlinear fitting analysis. The isotherm parameters of linear equations were determined from the relationships that are listed in Table 1. The presence of three parameters in the Redlich-Peterson and Dubinin-Astakhov equations required the optimization procedure of *K_RP_* and *n_DA_* parameters, respectively, in order to provide the maximum value of *r*^2^. It was carried out while using the solver add-in function with Microsoft^®^ Excel.

It should be pointed out that the conversion of nonlinear isotherm equations to linear forms for isotherm making alter their error structure [89]. Some authors recommend the usage of nonlinear method for the assessment of isotherm parameters rather than the use of correlation coefficient *r*^2^ of linear regression [89]. Therefore, alternatively to the linear regression, we also performed the estimation of isotherm parameters while using nonlinear fitting analysis. For finding out the most suitable parameters of nonlinear isotherm equations the Marquardt’s percent standard deviations (MPSD) error function was employed. The MPSD error function can be expressed as [84]:

(16) $$\mathrm{MPSD}=100\cdot \sqrt{\frac{1}{n-p}{\sum_{i=1}^{n}\left(\frac{Q_{e,\exp}-Q_{e,calc}}{Q_{e,\exp}}\right)}_{i}^{2}}$$

where *Q_e,exp_* (mg/g) and *Q_e,calc_* (mg/g) are the measured amount of adsorbed 18β-GA and calculated amount of adsorbed substance, respectively; *n* is the number of experimental points; and, *p* is the number of constants in the isotherm equation. 

The optimization procedure was performed by the minimization of MPSD error function values while using the solver add-in with Microscoft^®^ Excel Software.

### 2.6. Characterization Methods

Nitrogen adsorption–desorption experiments were conducted at −196 °C using an Autosorb iQ analyser (Quantachrome Instruments, Boynton Beach, FL, USA). The surface areas were determined from the Brunauer–Emmett–Teller (BET) equation. The pore size distribution, pore volume, and average pore diameter were calculated from the desorption branch of nitrogen isotherm based on the Barret–Joyner–Halenda (BJH) procedure. The thermogravimetric analysis (TGA) was carried out in a flow of air with a heating rate of 10 °C/min. from room temperature to 800 °C on a Setsys 1200 Setaram (Caluire, France) instrument. Transmission electron microscopy (TEM) micrographs were collected on a JOEL JEM 1200 EX (Tokyo, Japan) electron microscope operating at 80 kV. The Fourier-transform infrared (FT-IR) spectra were recorded with a Bruker FT-IR IFS 66 v/S (Karlsruhe, Germany) vacuum spectrometer in the wavelength range of 4000–400 cm^−1^ while using the KBr pellet technique. Spectrophotometric analyses were performed while using a Beckman DU 7500 (Fullerton, CA, USA) spectrophotometer.

## 3. Results and Discussion

### 3.1. Characterization of the Adsorbents

**Figure 2:**
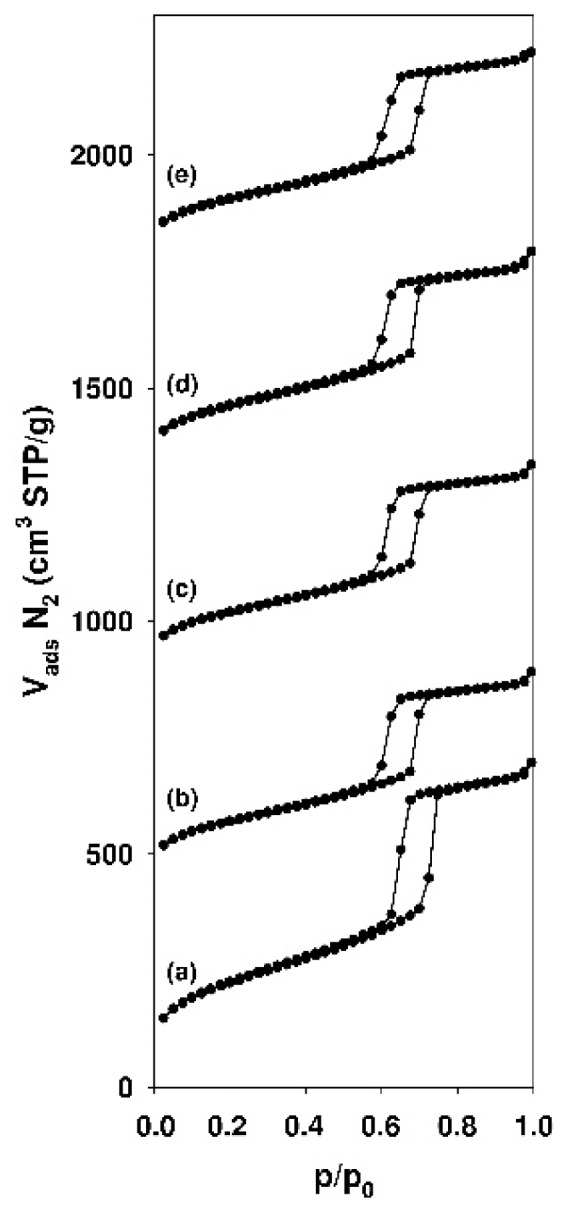
Nitrogen adsorption-desorption isotherms of (**a**) SBA-15, (**b**) SBA-15-AP, (**c**) SBA-15-MAP, (**d**) SBA-15-DMAP, and (**e**) SBA-15-AEAP mesoporous silicas. The isotherms were shifted from each other by 450 cm3/g along the Y-axis.

Figure 2 show the nitrogen adsorption-desorption isotherms for pure and functionalized SBA-15 silicas.

The corresponding textural properties of the adsorbents that were derived from this analysis are listed in Table 2. Pure and modified SBA-15 samples displayed typical type IV isotherm, according to IUPAC nomenclature [1], with the adsorption-desorption hysteresis loop characteristic for capillary condensation within uniform pores. All of the samples revealed a H1-type hysteresis loop, which is characteristic for a cylindrical-like pore structure. The isotherm reveals the sharp adsorption and desorption branches that were attributed to the narrow pore size distribution [90]. For functionalized SBA-15 samples, the nitrogen adsorption-desorption isotherms exhibit the similar shape and position of hysteresis loop with respect to non-modified material. However, the adsorbed nitrogen volume decreased and the slight flattening of the hysteresis loops can be observed as compared to parent silica. Parent and modified SBA-15 samples revealed a hysteresis loop at the relative pressure range from 0.60 to 0.75 and from 0.57 to 0.72, respectively.

The corresponding textural properties that were derived from the nitrogen sorption analysis for mesoporous SBA-15 samples and the BET surface analysis data for mesoporous and non-porous silicas are presented in Table 2. As compared to parent mesoporous samples, modified materials revealed reduced surface area, pore volume, and pore diameter values of about 43%–50%, 29%–31%, and 6.9%–8.6%, respectively. The decrease of surface parameter values depended on the type of used modifying agent and it was the most noticeable for mesoporous silica modified with AEAPTMS. Obtained results may confirm the anchorage of the organic groups onto the siliceous matrices. The introduced organic functions partially fill the pores and, therefore, also reduce in part the porosity of the samples [91]. The sorption analysis that was performed for pure non-porous commercial silica (Aerosil^®^) yielded a specific surface area of 181 m^2^/g. After the grafting process, the specific surface area of colloidal silica was reduced by 7.2%–17.7% as compared to parent material and it was the most meaningful for the sample modified with AEAPTMS. 

**Figure 3:**
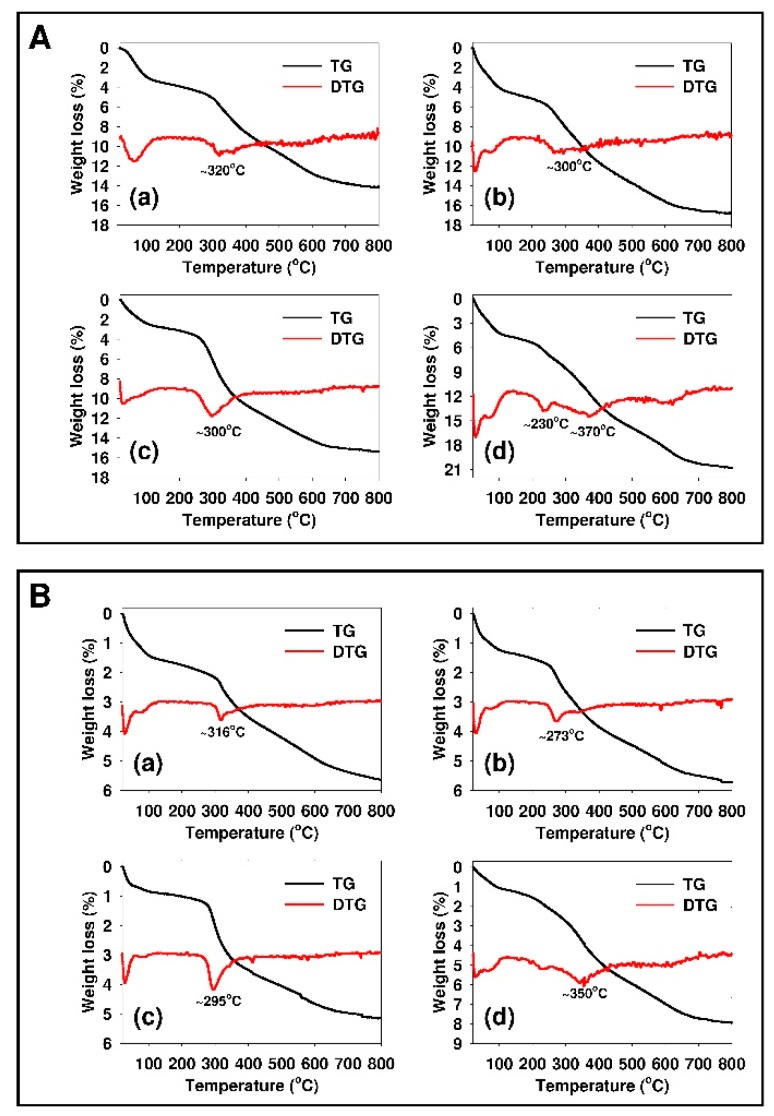
Thermogravimetric analysis of (**A**) functionalized SBA-15: (**a**) SBA-15-AP, (**b**) SBA-15-MAP, (**c**) SBA-15-DMAP, (**d**) SBA-15-AEAP, and (**B**) functionalized Aerosil^®^: (**a**) Aer-15-AP, (**b**) Aer-15-MAP, (**c**) Aer-15-DMAP, and (**d**) Aer-AEAP.

The results of thermogravimetric analysis confirmed the success of functionalization of siliceous adsorbents with organic moieties. Figure 3 shows the thermogravimetry (TG) and differential thermogravimetry (DTG) curves for modified mesoporous (see Figure 3A) and non-porous (see Figure 3B) silicas. 

The initial weight loss (minimum DTG value below 100 °C) can be mainly attributed to the desorption of physically adsorbed water [92]. Other substantial weight losses can be assigned to the decomposition of organic groups that were anchored at the siliceous surface (DTG minima for individual samples are indicated on the graph). The content of introduced organic moieties was calculated based on the weight losses observed at the temperature range from 200 to 650 °C for siliceous sorbents that were modified with APTMS, MAPTMS, and DMAPTMS. For AEAPTMS-modified samples, the decomposition temperature range was from 200 to 700 °C. The amount of incorporated organic functions was from 1.30 × 10^−3^ to 1.55 × 10^−3^ mol/g for SBA-15 mesoporous silica functionalized with DMAPTMS and APTMS, respectively. The content of functional groups that was calculated for non-porous silica was over twofold lower as compared to the SBA-15 sample. The amount of incorporated organic functions was from 4.32 × 10^−4^ to 6.10 × 10^−4^ mol/g for Aerosil^®^ that was modified with DMAPTMS and AEAPTMS, respectively.

**Figure 4:**
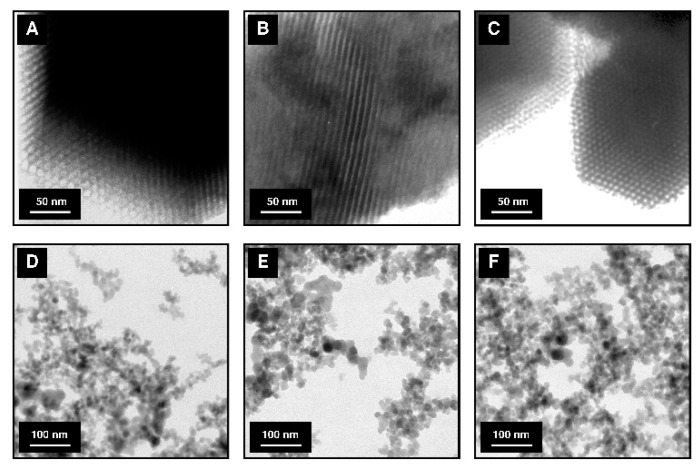
TEM micrographs of pure and modified siliceous adsorbents: (**A**) SBA-15, (**B**) SBA-15-AP, (**C**) SBA-15-MAP, (**D**) Aerosil^®^, (**E**) Aer-AP, and (**F**) Aer-MAP.

Figure 4 shows the results of transmission electron microscopy (TEM) analysis for mesoporous and non-porous siliceous sorbents.

The TEM micrograph of pure SBA-15 silica (see Figure 4A) revealed the hexagonal arrangement of mesoporous channels, which is in agreement with previous literature [93]. Figure 4B,C depict APTMS and MAPTMS-modified SBA-15 silicas characteristic for this structure parallel and hexagonal arrangement of mesoporous channels, respectively. The TEM micrographs for the functionalized mesoporous samples indicated that the surface modification process does not affect the siliceous structure. From the TEM micrograph of non-porous commercial silica (see Figure 4D), it can be noted that these particles are made of the aggregates of small spherical elementary particles with the diameter between ten and twenty nanometers [94]. A similar morphology can be distinguished in TEM micrographs for APTMS and MAPTMS-functionalized Aerosil^®^ (see Figure 4E,F respectively).

**Figure 5:**
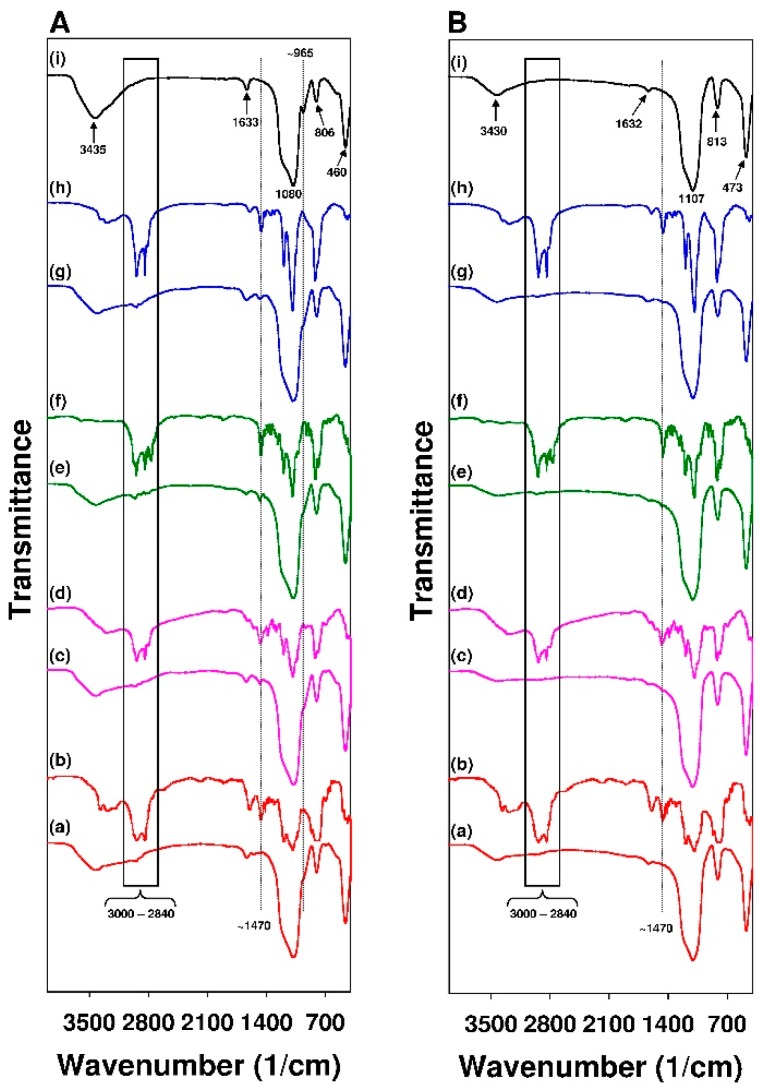
FT-IR spectra of (**A**) SBA-15 silicas and adequate modifying agents: (**a**) SBA-15-AP, (**b**) APTMS, (**c**) SBA-15-MAP, (**d**) MAPTMS, (**e**) SBA-15-DMAP, (**f**) DMAPTMS, (**g**) SBA-15-AEAP, (**h**) AEAPTMS, (**i**) pure SBA-15 silica; (**B**) Aerosil^®^ and adequate modyfying agents: (**a**) Aer-15-AP, (**b**) APTMS, (**c**) Aer-15-MAP, (**d**) MAPTMS, (**e**) Aer-DMAP, (**f**) DMAPTMS, (**g**) Aer-AEAP, (**h**) AEAPTMS, and (**i**) pure Aerosil^®^.

The presence of organic functions that were introduced on the siliceous surface was further confirmed while using Fourier-transform infrared (FT-IR) spectroscopy. The FT-IR spectra of mesoporous and non-porous sorbents are shown in Figure 5A,B respectively.

The spectra of modified SBA-15 samples (see Figure 5A) revealed several absorption bands that were located in the range from 3000 to 2840 cm^−1^ that can be ascribed to the C-H stretching vibrations [95] of alkyl chains of the introduced functional groups. Moreover, the strong absorption band with the maximum localized at around 1470 cm^−1^ can be assigned to the bending vibrations (scissoring) of the -CH_2_ group [95] (for easier comparison the spectra of individual modifying agents are also presented). In the FT-IR spectra of mesoporous sorbents, the absorption bands that were located at around 3435, 1633, 1081, 965, 806, and 460 cm^−1^ ascribed to the absorption of infrared radiation by the silica material [41,96] can be distinguished. It is also worth mentioning that, in the spectra of modified SBA-15 samples, the vibrational band localized at 965 cm^−1^ assigned to the stretching mode of the Si-OH group disappeared. It can further confirm the successful modification of mesoporous materials [96]. The FT-IR spectra of functionalized Aerosil^®^ (see Figure 5B) revealed considerably weaker absorption bands that were assigned to the presence of alkyl chains as compared to modified SBA-15 samples. It might result from the lower content of these functional groups in Aerosil^®^, which was confirmed by the results of TG analysis. Similarly, a weaker absorption band with the maximum localized at around 1470 cm^−1^ ascribed to bending vibrations (scissoring) of –CH_2_ group can be distinguished.

### 3.2. Adsorption Studies

**Figure 6:**
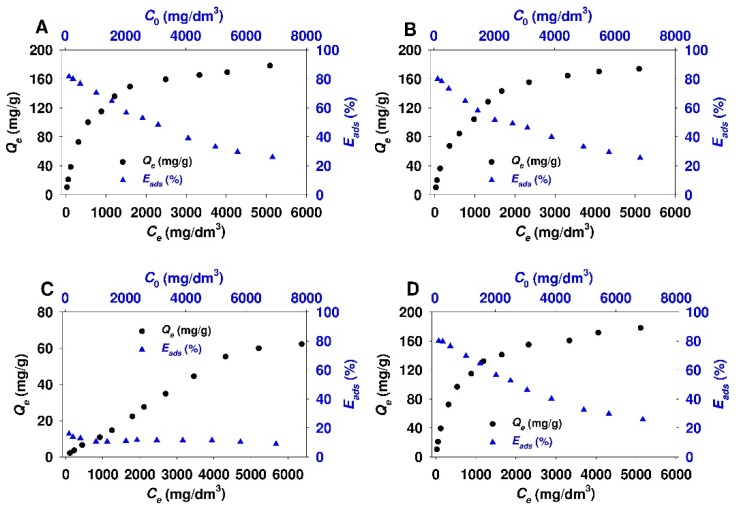
Adsorption isotherms and adsorption efficiency of 18β-glycyrrhetinic acid onto: (**A**) SBA-15-AP, (**B**) SBA-15-MAP, (**C**) SBA-15-DMAP, and (**D**) SBA-15-AEAP mesoporous sorbents.

**Figure 7:**
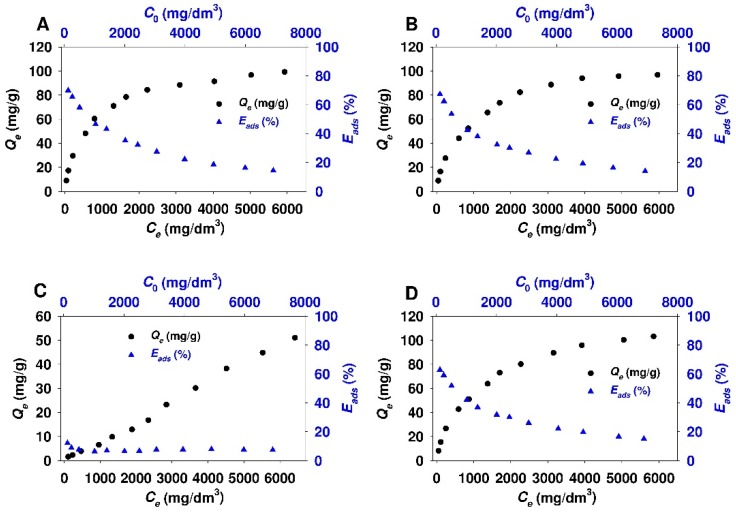
Adsorption isotherms and adsorption efficiency of 18β-glycyrrhetinic acid onto: (**A**) Aer-AP, (**B**) Aer-15-MAP, (**C**) Aer-DMAP, and (**D**) Aer-AEAP colloidal silicas.

The results of the adsorption studies of 18β-GA acid onto functionalized mesoporous and non-porous siliceous adsorbents are demonstrated in Figure 6 and Figure 7, respectively.

For all SBA-15 and Aerosil^®^ samples that were functionalized with APTMS, MAPTMS, and AEAPTMS, the two-phase isotherm profile characteristic for the Langmuir-type adsorption isotherm with the sharp initial slope at lower equilibrium adsorbate concentration (C_e_ < 2000 mg/dm^3^), followed by a plateau at higher equilibrium 18β-GA concentration, can be distinguished. In the case of Aer-AEA sample, the plateau is less visible and it appears at higher equilibrium adsorbate concentration (> 4500 mg/dm^3^). Interestingly, for both sorbents that were modified with N,N-dimethylaminopropyl groups, the linear relationship between the amount of the adsorbate and its equilibrium concentration almost at all equilibrium concentration range can be observed. This type of the isotherm indicates the constant partition of the adsorbate between the solution and the adsorbent. It is the so-called the C isotherm curve, according to Giles et al. classification [97]. The shape of adsorption isotherm and the position of plateau show that SBA-15 sorbents that were modified with APTMS, MAPTMS, and AEAPTMS are characterized by the comparable adsorption capacity (~160 mg/g). A similar trend was observed for Aerosil^®^ samples functionalized with above-mentioned modifying agents. For colloidal silicas, the adsorption capacity reaches ~100 mg/g. The adsorption efficiency for SBA-15-AP, SBA-15-MAP, and SBA-15-AEAP sorbents decreased with the increase of equilibrium adsorbate concentration. It was in the range from ~80 to ~25% for initial adsorbate concentration of 120 mg/dm^3^ and 6900 mg/dm^3^, respectively. The adsorption efficiency for colloidal silica modified with the same functional groups was in the range from ~65 to ~14% for respective initial 18β-GA concentrations. SBA-15-DMAP and Aer-DMAP samples both revealed low adsorption efficiency almost at all initial adsorbate concentration range that did not exceed a value between ten and twenty per cent. 

**Figure 8:**
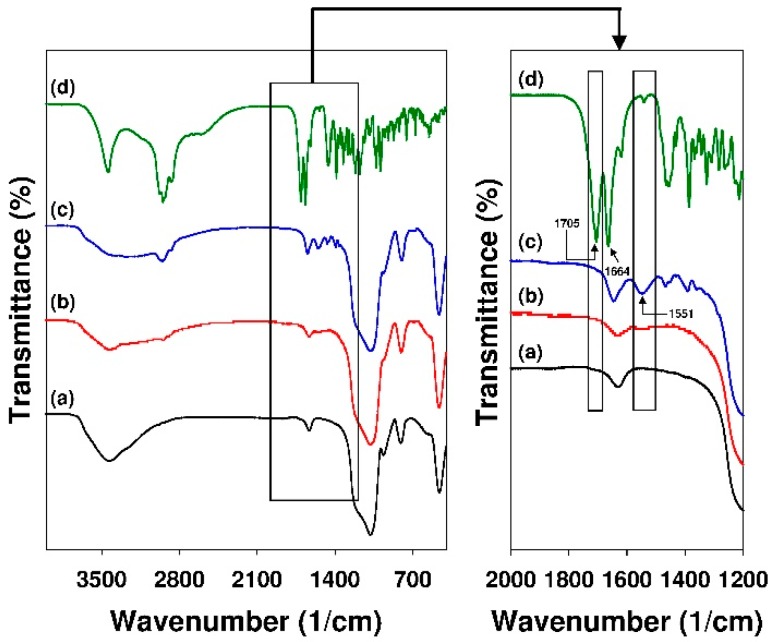
FT-IR spectra of (**a**) pure SBA-15, (**b**) SBA-15-AP, (**c**) SBA-15-AP+18β-GA, (**d**) 18β-glycyrrhetinic acid (18β-GA). The picture at the right shows details of the spectra.

Figure 8 presents the FT-IR spectrum of SBA-15-AP sample with the adsorbed 18β-GA. For easier comparison, the spectra of unmodified mesoporous sorbent and pure 18β-GA are provided.

The results of the analysis revealed the absence of an absorption band located at 1705 cm^−1^ ascribed to the stretching vibrations of 18β-GA carboxyl group [98,99] (compare spectra c and d). Furthermore, the new absorption band at 1551 cm^−1^ assigned to the stretching vibrations of COO^−^ group can be distinguished [98]. It might confirm the ionization of 18β-GA adsorbed onto the SBA-15-AP surface. This band is not observed in the spectra of pure silica and its modified form (see spectra a and b, respectively). In the FT-IR spectrum of 18β-GA, a strong absorption band located at 1664 cm^−1^ ascribed to its conjugated carbonyl groups (carbon C11) can be noticed [99]. The adsorption band at 1667 cm^−1^ confirming the presence of conjugated carbonyl groups of 18β-GA is also observed in the spectrum of SBA-15-AP sample with adsorbed 18β-GA. This band partially overlaps with the absorption band of silica itself; however, for the SBA-15-AP+GA sample, it is more intensive.

### 3.3. Estimation of Isotherm Parameters Using Linear Regression

The experimental data of 18β-GA adsorption onto modified siliceous sorbents (see Figure 6 and Figure 7) were analysed while using several adsorption models. The parameters of adsorption isotherms that were calculated from the linear regression based on the Equations (9)–(14) for functionalized SBA-15 and Aerosil^®^ samples are shown in Table 3 and Table 4, respectively. As can be seen from the presented data, the Dubinin-Astakhov, Redlich-Peterson, and Langmuir isotherms described the best (r^2^ ~0.99) the process of adsorption of 18β-GA onto mesoporous and colloidal silicas that were modified with APTMS, MAPTMS, and AEAPTMS. Meanwhile, the Freundlich model best describes the process of 18β-GA adsorption on SBA-15-DMAP and Aer-DMAP samples containing tertiary amine group. The analysis of the maximum adsorption capacity of modified siliceous sorbents towards 18β-GA revealed twice better adsorption capacity of SBA-15 silicas as compared to the modified colloidal sorbents. As an example, the adsorption capacity of SBA-15-AP sample was in the range from 169.5 to 286.3 mg/g, depending on the model used, whereas for Aer-AP sorbent, this value was in the range from 89.3 to 144.9 mg/g. For the specified type of silica functionalized with APTMS, MAPTMS, and AEAPTMS, the comparable adsorption capacity was noted. The highest values of Langmuir constant (K_L_) were observed for SBA-15-AP, SBA-15-MAP, Aer-AP, and Aer-MAP silicas. For adsorbents that were modified with ethylenediamine derivative, these values were insignificantly lower. For the DMAPTMS-modified SBA-15 sample, the values of the K_L_ parameter were one order of magnitude lower when compared to other adsorbents. The values of the β parameter determined from the Redlich-Peterson equation were in the range from 0.78 to 0.93 and from 0.74 to 0.82 for APTMS, MAPTMS, and AEAPTMS- functionalized SBA-15 and Aerosil^®^ samples, respectively. It might indicate that this type of adsorption is consistent with the Langmuir model [87]. The adsorption isotherms that are based on the Polanyi potential enable the determination of mean free energy of adsorption [82,83,85], as follows:

(17) $$E_{DR}=\frac{1}{\sqrt{2\cdot K_{DR}}}$$

(18) $$E_{DA}=\frac{1}{\sqrt{2}\cdot \sqrt[{n_{DA}}]{K_{DA}}}$$

where *E_DR_* and *E_DA_* represent the adsorption energy (J/mol) calculated from Dubinin–Radushkevich and Dubinin–Astakhov isotherms, respectively; *K_DR_* (mol^2^/J^2^) and *K_DA_* (mol^nDA^/J^nDA^) describe the constant related to the energy of adsorption for given isotherms; *n_DA_* indicates the heterogeneity factor appearing in the Dubinin–Astakhov equation.

**Figure 9:**
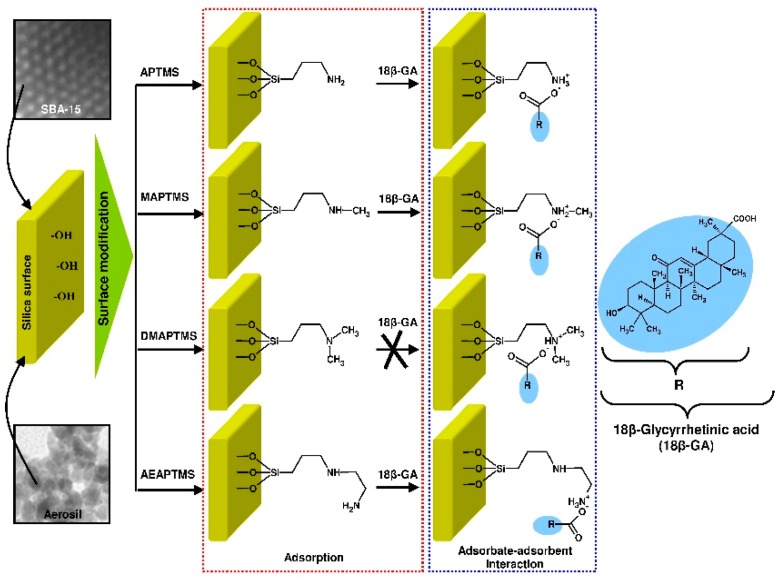
The scheme presenting interactions between 18β-glycyrrhetinic acid and aminopropyl-derivative-modified siliceous surface.

The values of adsorption energy of 18β-GA calculated from the Dubinin–Radushkevich equation were in the range from 5.56 to 7.85 and from 5.51 to 8.00 kJ/mol for trialkoxysilane-modified SBA-15 and Aerosil^®^ silicas, respectively. However, the lowest values of ~5.5 kJ/mol were noted for the adsorption of 18β-GA onto silicas functionalized with DMAPTMS. It unambiguously indicates the physical nature [100] of interactions between the adsorbate and the siliceous surface modified with tertiary amine derivative. The calculated values of adsorption energy also correspond with the Freundlich model that describes the best the process of 18β-GA adsorption onto SBA-15-DMAP and Aer-DMAP sorbents. For silicas that were modified with APTMS, MAPTMS, and AEAPTMS, these values are similar and approach the limit value of 8 kJ/mol. This energy value differentiates physical from chemical adsorption [100,101]. The adsorption energy calculated from the Dubinin–Astakhov model revealed slightly higher values as compared to those that were established from the Dubinin–Radushkevich equation and was in the range from 8.35 to 9.45 and from 7.69 to 9.04 kJ/mol for SBA-15 and Aerosil^®^ samples, respectively. These values should be considered as being more suitable due to better fit (higher r^2^ values) of the Dubinin–Astakhov model as compared to Dubinin-Radushkevich one. The values of adsorption energy determined from Dubinin–Astakhov equation for SBA-15-AP, SBA-15-MAP, Aer-AP, and Aer-MAP sorbents unequivocally indicate the chemical nature of interactions [102] and may suggest the formation of ionic pairs between the 18β-GA and sorbent amine groups. The possibility of such interactions seems to be confirmed by the results of previously described FT-IR analysis regarding the SBA-15-AP sorbent with adsorbed 18β-GA. Figure 9 shows the schematic representation of possible interactions between 18β-GA and modified siliceous surface.

**Figure 10:**
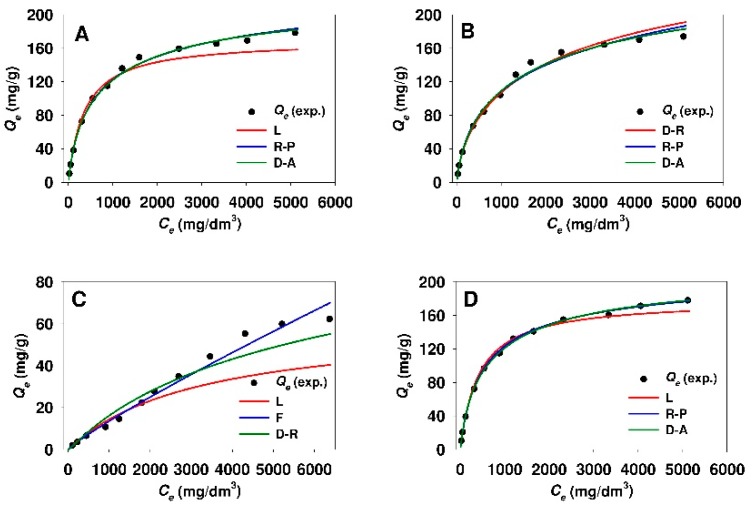
Comparison of experimental and predicted isotherms of 18β-glycyrrhetinic acid adsorption onto (**A**) SBA-15-AP, (**B**) SBA-15-MAP, (**C**) SBA-15-DMAP, and (**D**) SBA-15-AEAP mesoporous adsorbents. The best fitted isotherm models derived from linear analysis are presented. Isotherm models: F, Freundlich; D-A, Dubinin-Astakhov; D-R, Dubinin-Radushkevich; L, Langmuir; R-P, Redlich-Peterson.

**Figure 11:**
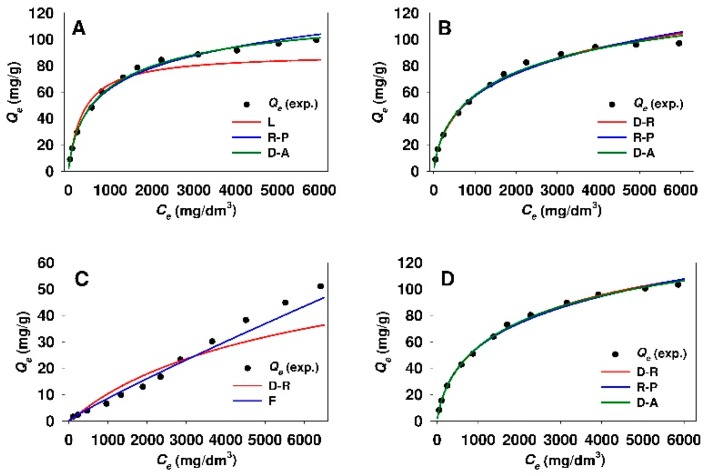
Comparison of experimental and predicted isotherms of 18β-glycyrrhetinic acid adsorption onto (**A**) Aer-AP, (**B**) Aer-MAP, (**C**) Aer-DMAP, and (**D**) Aer-AEAP adsorbents. The best fitted isotherm models derived from linear analysis are presented. Isotherm models: F, Freundlich; D-A, Dubinin-Astakhov; D-R, Dubinin-Radushkevich; L, Langmuir; R-P, Redlich-Peterson.

The values of n_DA_ heterogeneity parameter calculated from the Dubinin–Astakhov model were insignificantly lower for the adsorption of 18β-GA onto modified Aerosil^®^ silica as compared to functionalized SBA-15 sample. Figure 10 and Figure 11 demonstrate the comparison of experimental and predicted adsorption isotherms, where the parameters were established from linear regression for modified mesoporous and non-porous siliceous sorbents, respectively.

The curves would seem to suggest that, in the case of both silicas modified with APTMS, MAPTMS, and AEAPTMS, the adsorption models that are based on the Polanyi potential and the Redlich-Peterson model are well fitted at the whole equilibrium adsorbate concentration range. Meanwhile, the Freundlich equation evidences the best fit for SBA-15-DMAP and Aer-DMAP sorbents.

### 3.4. Estimation of Isotherm Parameters Using Nonlinear Fitting Analysis

**Figure 12:**
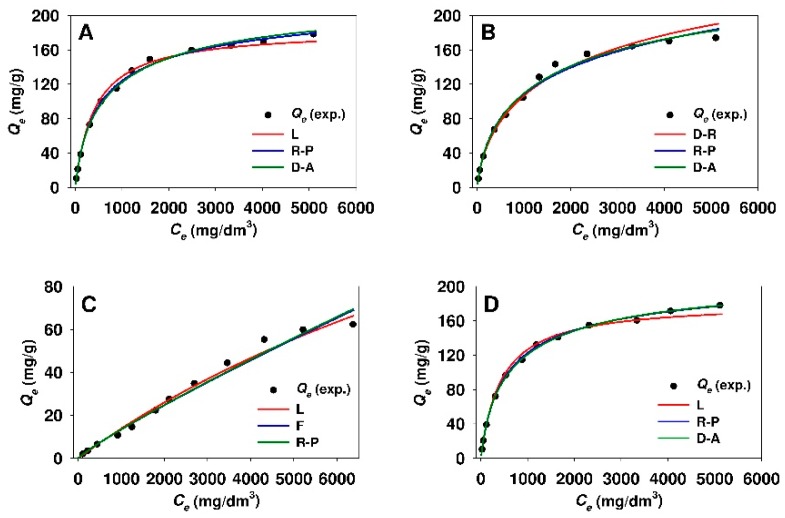
Comparison of experimental and predicted isotherms of 18β-glycyrrhetinic acid adsorption onto (**A**) SBA-15-AP, (**B**) SBA-15-MAP, (**C**) SBA-15-DMAP, and (**D**) SBA-15-AEAP mesoporous adsorbents. The best fitted isotherm models derived from nonlinear fitting analysis are presented. Isotherm models: F, Freundlich; D-A, Dubinin-Astakhov; D-R, Dubinin-Radushkevich; L, Langmuir; R-P, Redlich-Peterson.

**Figure 13:**
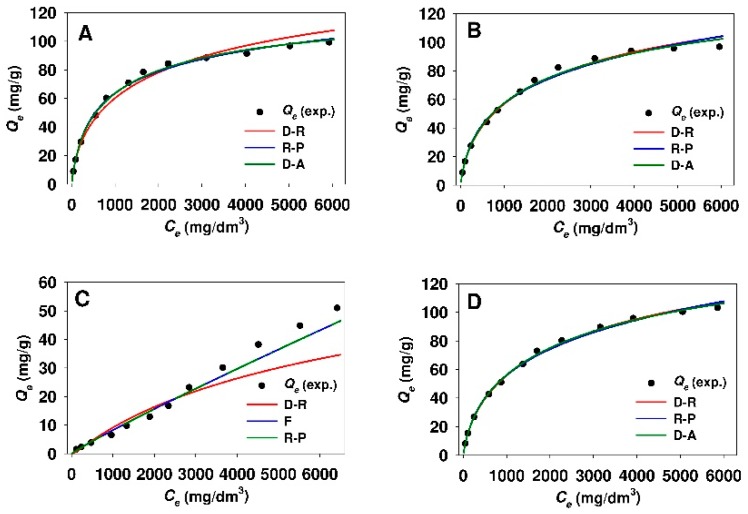
Comparison of experimental and predicted isotherms of 18β-glycyrrhetinic acid adsorption onto (**A**) Aer-AP, (**B**) Aer-MAP, (**C**) Aer-DMAP, and (**D**) Aer-AEAP adsorbents. The best fitted isotherm models derived from nonlinear fitting analysis are presented. Isotherm models: F, Freundlich; D-A, Dubinin-Astakhov; D-R, Dubinin-Radushkevich; R-P, Redlich-Peterson.

The nonlinear fitting analysis was also applied in order to obtain the optimum isotherm parameters. Table 5 and Table 6 provide the values of the parameters determined from Equations (3)–(8) while using the MPSD error function for the adsorption of 18β-GA onto modified mesoporous and non-porous siliceous materials, respectively. While taking the minimized values of MSPD error function into account, it can be concluded that the Dubinin-Astakhov, Dubinin-Radushkevich, and Redlich-Peterson models revealed the best fit of the isotherms to the experimental points for the adsorption of 18β-GA onto both sorbents that were modified with APTMS, MAPTMS, and AEAPTMS. For these isotherms, the value of MPSD error function was in the range from 1.99 to 10.16 and from 2.27 to 6.31 for functionalized SBA-15 and Aerosil^®^ samples, respectively. Whereas, the adsorption of 18β-GA onto DMAP-functionalized silicas is best described by the Freundlich model. The comparison of the experimental and predicted adsorption isotherms (for three best fitted adsorption models) where the parameters were assessed from nonlinear fitting analysis are presented in Figure 12 and Figure 13 for modified SBA-15 and Aerosil^®^ silicas, respectively. 

While taking into consideration different methodologies of isotherm parameter estimation the list and sequence of isotherm fit to the experimental data is presented in Table 7. Based on the values of correlation coefficient r^2^ and MPSD error function, it can be concluded that the order of isotherm fit is similar to the one that arises from linear regression. The values of Q_L(max)_ parameter for both sorbents that were modified with APTMS, MAPTMS, and AEAPTMS established from nonlinear fitting analysis were higher when compared to the values that were derived from linear regression. Namely, the Q_L(max)_ values were of 2.0% to 16.6% and of 11.5% to 16.8% higher for modified SBA-15 and Aerosil^®^ silicas, respectively, as compared to the values that were calculated from linear regression for respective functionalized silicas. For particular adsorbents, the maximum adsorption capacity Q_DR(max)_ and Q_DA(max)_ estimated from linear regression and nonlinear fitting analysis revealed similar values.

The mean energy of 18β-GA adsorption onto SBA-15 silica modified with APTMS, MAPTMS, and AEAPTMS determined from the Dubinin–Astakhov and Dubinin–Radushkevich isotherms was in the range from 7.73 to 7.85 kJ/mol and from 8.37 to 9.44 kJ/mol, respectively. In the case of non-porous sorbent modified while using the same functional groups, the E_DA_ and E_DR_ values were in the range from 7.70 to 9.03 and from 7.53 to 8.00 kJ/mol, respectively.

While taking the maximum adsorption capacity (Q_ads(max)_, mg/g) established from the Langmuir, Dubinin–Radushkevich, and Dubinin–Astakhov model into account, the value of molar ratio between the amount of adsorbed 18β-GA to the content of functional groups of the individual adsorbent (Q_FG_) was as follows:

(19) $$\frac{n_{\mathrm{GA}}}{n_{\mathrm{FG}}}=\frac{Q_{ads(\max)}\cdot 10^{-3}}{Q_{\mathrm{FG}}\cdot M_{\mathrm{GA}}}$$

where n_GA_ and n_FG_ represent the number of moles of 18β-GA and aminopropyl derivative functional groups, respectively; M_GA_ is the molar weight (mol/g) of 18β-GA.

**Figure 14:**
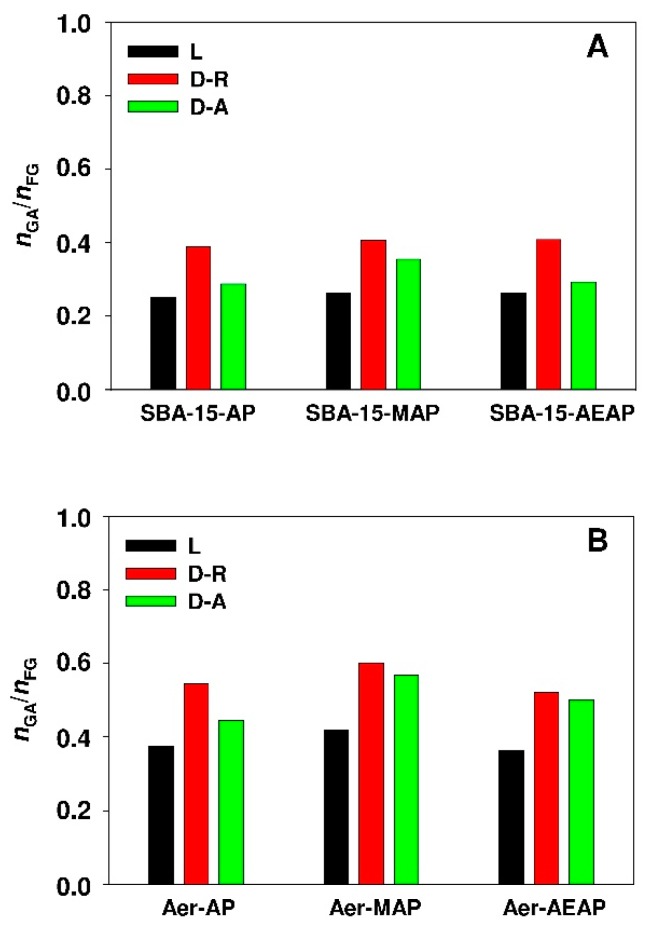
Molar ratio of 18β-glycyrrhetinic acid molecules to functional group content of (**A**) modified SBA-15 silicas and (**B**) modified Aerosil^®^ siliacas. The ratio calculated from maximum adsorption capacity established from nonlinear fitting analysis. Isotherms: D-A, Dubinin-Astakhov; D-R, Dubinin-Radushkevich; L, Langmuir.

The results of the above-mentioned calculations established on the basis of Q_ads(max)_ parameter for particular isotherms while using nonlinear fitting analysis are demonstrated in Figure 14.

In view of presented data, it can be clearly seen that the value of n_GA_/n_FG_ molar ratio for the relevant sorbents is higher for the modified non-porous samples (Figure 14B) as compared to functionalized mesoporous silicas (Figure 14A). The relationship might be explained by the greater availability of adsorption sites of non-porous sample as compared with nanoporous SBA-15 silica. It seems that, in the case of SBA-15 sorbent, the adsorption limiting factor is the restricted mesoporous size, especially since the content of SBA-15 functional groups is more than twice over colloidal silica (see Table 2). The highest value of the n_GA_/n_FG_ molar ratio was reported for Aerosil^®^ modified with MAPTMS. It is worth noting that the values of n_GA_/n_FG_ molar ratio depend on employed calculation model and they are significantly lower than the unity. This is an indication that basic adsorption sites are partially available. Such a low degree of accessibility was also observed for the adsorption of caffeic acid onto SBA-15 and MCF sorbents that were modified with 3-aminopropyl groups [48] or boldine alkaloid onto propyl-sulfonic acid-modified mesoporous silicas [49]. 

**Figure 15:**
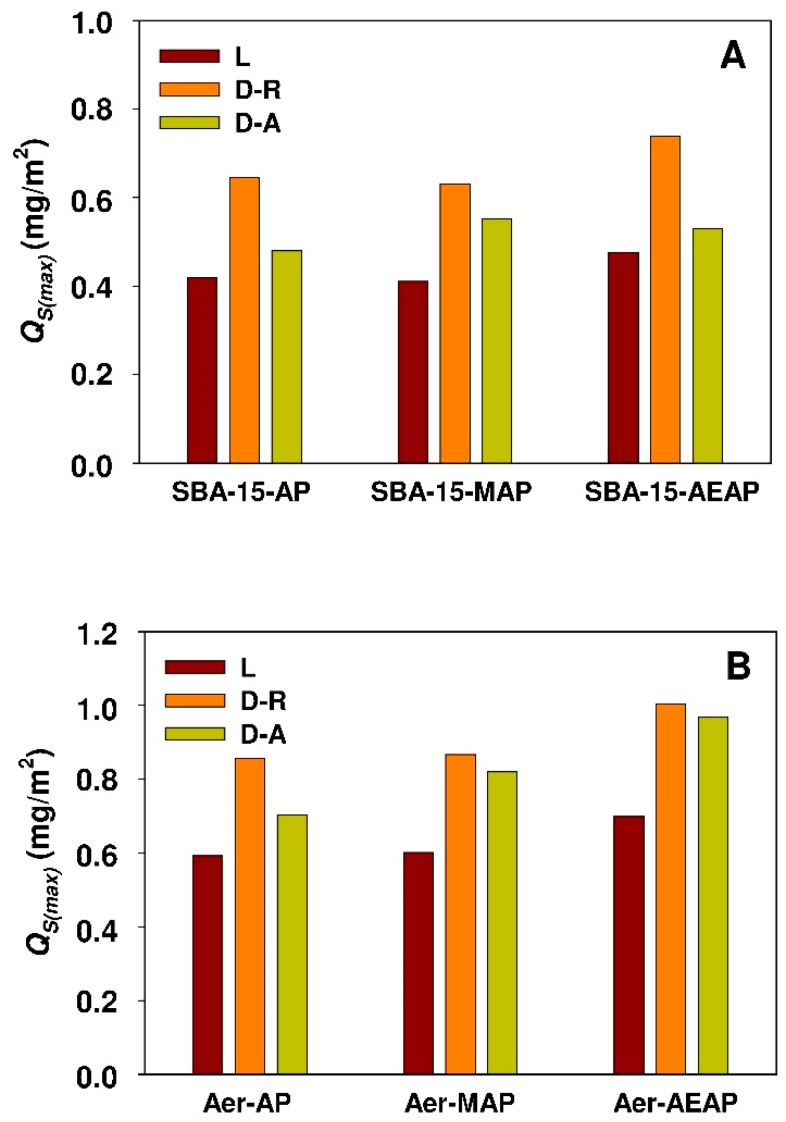
Surface area-normalized maximum adsorption capacity of (**A**) modified SBA-15 silicas and (**B**) modified Aerosil^®^ siliacas towards 18β-glycyrrhetinic acid. The parameter calculated from maximum adsorption capacity estimated from nonlinear fitting analysis. Isotherms: D-A, Dubinin-Astakhov; D-R, Dubinin-Radushkevich; L, Langmuir.

The phenomenon of worse accessibility of adsorption sites of mesoporous silicas appears to be consistent with the values of surface area-normalized adsorption capacity (*Q_s(max)_*, mg/m^2^) of modified SBA-15 and Aerosil^®^ sorbents that are presented in Figure 15. The values of *Q_s(max)_* parameter were calculated while using the following equation:

(20) $$Q_{S(\max)}=\frac{Q_{ads(\max)}}{S_{\mathrm{BET}}}$$

where *S_BET_* (m^2^/g) represents the specific surface area of siliceous adsorbents (see Table 2).

The underlying calculations revealed higher values of the *Q_s(max)_* parameter for modified non-porous silicas as compared to respective functionalized mesoporous sorbents (see Figure 15). 

It might indicate a better exploitation of surface adsorption sites of modified non-porous adsorbent. Similar results were achieved during comparative studies concerning the adsorption of chlorhexidine [41] onto non-modified Aerosil^®^ and few selected mesoporous silicas. In the case of 18β-GA adsorption, the highest value of Q_s(max)_ parameter was observed for Aerosil^®^ silica modified with ethylenediamine derivative (AEAPTMS) that simultaneously contains the primary and secondary amine group.

## 4. Conclusions

Given the variety of adsorbents and isotherm models that were used for description of 18β-GA adsorption process, for reasons of clarity, the main conclusions of this study regarding the optimization of isotherm parameters using linear regression and nonlinear fitting analysis are as follows:

1. The adsorption isotherms of 18β-GA onto silicas functionalized with APTMS, MAPTMS and AEAPTMS indicate the Langmuir-type adsorption, whereas sorbents that were modified with DMAPTMS show constant distribution of the adsorbate between the adsorbent and the solution regardless of silica type.
2. The Dubinin–Astakhov, Dubinin–Radushkevich, and Redlich–Peterson equations described the best the process of 18β-GA adsorption onto SBA-15 and Aerosil^®^ silicas functionalized with APTMS, MAPTMS, and AEAPTMS regardless of the method used for estimation of isotherm parameters (linear regression or nonlinear fitting analysis).
3. Based on nonlinear fitting analysis (Dubinin–Astakhov model), it can be concluded that SBA-15 sorbent modified with APTMS, MAPTMS, and AEAPTMS is characterized by twice the adsorption capacity (202.8–237.3 mg/g) as compared to functionalized Aerosil^®^ (118.2–144.2 mg/g).
4. The process of 18β-GA adsorption onto SBA-15 and Aerosil^®^ silicas that were modified with DMAPTMS is best described by the Freundlich model.
5. The Temkin isotherm is not suitable for the description of 18β-GA adsorption onto any of the used sorbents, owing to low *r*^2^ values (linear regression) or high values of MPSD error function (nonlinear fitting analysis).
6. The values of mean adsorption energy (Dubinin–Astakhov model) and analysis of FT-IR spectra revealed the chemical nature of interactions between 18β-GA and siliceous surface modified with APTMS, MAPTMS, and AEAPTMS, meanwhile the adsorption of 18β-GA onto silicas that were modified with DMAPTMS has a physical nature (Dubinin-Radushkevich model).
7. Higher values of molar ratio of the adsorbate to the sorbent functional groups and a higher value of surface area-normalized adsorption capacity for modified Aerosil^®^ silica demonstrate the better exploitation of adsorption sites of non-porous sorbent when compared to the SBA-15 sample.
8. The obtained adsorbents (SBA-15-AP, SBA-15-MAP, and SBA-15-AEAP) were characterized by the adsorption efficiency of 80% at the conditions of the lowest initial 18β-GA concentration (120 mg/dm^3^). For modified colloidal silicas, the adsorption efficiency reached 64%. The obtained results indicate that the SBA-15 material modified with trialkoxysilanes containing various amine groups (apart from the sample modified using (*N,N*-dimethylaminopropyl)trimethoxysilane)) is quite good adsorbent for 18β-GA. Previous studies that were also conducted in 2-propanol revealed better adsorption efficiency exceeding 90% for adsorption of carboxylic acids onto the surface of SBA-15 silica modified with 3-aminopropyl groups. It should be noted that examined adsorbates, such as diflunisal [38], caffeic acid [48], rosmarinic acid [103], and sinapic acid [104], are characterized by a slightly lower molar mass as compared to 18β-GA.

From the performed experiments, it can be concluded that the SBA-15 silicas modified with 3-(aminopropyl)trimethoxysilane (APTMS), [3-(methylamino)propyl]trimethoxysilane (MAPTMS), and [3-(2-aminoethylamino)propyl]trimethoxysialne (AEAPTMS) revealed significant adsorption capacity towards 18β-GA, whereas Aerosil^®^ sorbent that was functionalized while using the same modifying agents exhibited a better availability of adsorption sites towards the adsorbate. Silicas that were modified with (*N,N*-dimethylaminopropyl)trimethoxysilane (DMAPTMS) are characterized by both low adsorption capacity and adsorption efficiency.

## Figures and Tables

**Table 1:** Nonlinear and linear representation of adsorption isotherms.

Isotherm Model	Non-Linear Expression		Linear Transform	
			Equation	
Langmuir	$Q_{e}=\frac{Q_{L(\max)}\cdot K_{L}\cdot C_{e}}{1+K_{L}\cdot C_{e}}$	(3)	$\frac{1}{Q_{e}}=\frac{1}{Q_{L(\max)}\cdot K_{L}}\cdot \frac{1}{C_{e}}+\frac{1}{Q_{L(\max)}}$	(9)
Freundlich	$Q_{e}=K_{F}\cdot C^{1/n_{F}}$	(4)	$\ln Q_{e}=\frac{1}{n_{F}}\ln C_{e}+\ln K_{F}$	(10)
Redlich-Peterson	$Q_{e}=\frac{K_{RP}\cdot C_{e}}{1+a_{RP}\cdot C_{e}{}^{\beta }}$	(5)	$\ln\left(K_{RP}\cdot \frac{C_{e}}{Q_{e}}-1\right)=\beta \ln C_{e}+\ln a_{RP}$	(11)
Temkin	$Q_{e}=\frac{RT}{b_{T}}\ln(K_{T}\cdot C_{e})$	(6)	$Q_{e}=\frac{RT}{b_{T}}\ln K_{T}+\frac{RT}{b_{T}}\ln C_{e}$	(12)
Dubinin-Radushkevich	$Q_{e}=Q_{DR(\max)}\exp\left\{-K_{DR}{\left[RT\ln\left(\frac{C_{s}}{C_{e}}\right)\right]}^{2}\right\}$	(7)	$\ln Q_{e}=-K_{DR}{\left[RT\ln\left(\frac{C_{s}}{C_{e}}\right)\right]}^{2}+\ln Q_{DR(\max)}$	(13)
Dubinin-Astakhov	$Q_{e}=Q_{DA(\max)}\exp\left\{-K_{DA}{\left[RT\ln\left(\frac{C_{s}}{C_{e}}\right)\right]}^{n_{DA}}\right\}$	(8)	$\ln Q_{e}=-K_{DA}{\left[RT\ln\left(\frac{C_{s}}{C_{e}}\right)\right]}^{n_{DA}}+\ln Q_{DA(\max)}$	(14)

Description of symbols: *a_RP_*: Redlich-Peterson constant (dm^3β^/mg^β^); *b_T_*: Temkin constant related to the adsorption heat (J g/mol mg); *β*: exponential constant of Redlich-Peterson isotherm; *C_e_*: equilibrium concentration of adsorbate (mg/dm^3^); *C_s_*: solubility of adsorbate (mg/dm^3^); *K_DA_*: Dubinin-Astakhov isotherm constant related to the sorption energy (mol*^nDA^*/J*^nDA^*); *K_DR_*: Dubinin-Radushkevich isotherm constant related to the sorption energy (mol^2^/J^2^); *K_F_*: Freundlich constant (mg^1-1/n^dm^3/n^/g); *K_L_*: Langmuir constant (dm^3^/mg); *K_RP_*: Redlich-Peterson constant (dm^3^/g); *K_T_*: Temkin equilibrium binding constant (dm^3^/mg); *n_DA_*: heterogeneity factor of Dubinin-Astakhov isotherm; *n_F_*: exponential constant of Freundlich isotherm; *Q_DA(max)_*: maximum adsorption capacity estimated from Dubinin-Astakhov model (mg/g); *Q_DR(max)_*: maximum adsorption capacity calculated from Dubinin-Radushkevich model (mg/g); *Q_e_*: equilibrium amount of adsorbate (mg/g); *Q_L(max)_*: maximum adsorption capacity calculated from Langmuir model (mg/g); *R*: gas constant (8.314 J/ mol K); *T*: absolute temperature (K).

**Table 2:** Textural properties of siliceous adsorbents.

Adsorbent	Modifying Agent	Amount of Functional Groups, *Q_FG_* (mol/g) ^a^	BET Surface Area (m^2^/g)	BJH Pore Volume (cm^3^/g) ^b^	Pore Diameter (nm) ^b^
SBA-15	−	−	770	0.96	5.8
SBA-15-AP	APTMS	1.55 × 10^−3^	438	0.67	5.4
SBA-15-MAP	MAPTMS	1.42 × 10^−3^	430	0.67	5.4
SBA-15-DMAP	DMAPTMS	1.30 × 10^−3^	425	0.68	5.4
SBA-15-AEAP	AEAPTMS	1.47 × 10^−3^	382	0.66	5.3
Aer	−	−	181	−	−
Aer-AP	APTMS	5.63 × 10^−4^	168	−	−
Aer-MAP	MAPTMS	4.99 × 10^−4^	163	−	−
Aer-DMAP	DMAPTMS	4.32 × 10^−4^	158	−	−
Aer-AEAP	AEAPTMS	6.10 × 10^−4^	149	−	−

Abbreviations: APTMS, (3-aminopropyl)trimethoxysilane; MAPTMS, [3-(methylamino)propyl]-trimethoxysilane; DMAPTMS, (*N,N*-dimethylaminopropyl)trimethoxysilane; AEAPTMS, [3-(2-aminoethylamino)propyl]trimethoxysilane. ^a^ Calculated from thermogravimetric analysis. ^b^ Calculated from desorption branch of isotherm.

**Table 3:** Isotherm parameters calculated from linear regression for 18β-glycyrrhetinic acid adsorption onto mesoporous silica functionalized with various amine groups.

Adsorption model	Parameter	Adsorbent			
		SBA-15-AP	SBA-15-MAP	SBA-15-DMAP	SBA-15-AEAP
Langmuir	*Q_L(max)_* (mg/g)	169.5	151.5	61.0	178.6
	*K_L_* (dm^3^/mg)	2.745 × 10^−3^	2.794 × 10^−3^	3.072 × 10^−4^	2.384 × 10^−3^
	*r* ^2^	0.9992	0.9970	0.9903	0.9993
Freundlich	*K_F_* (mg^1−1/n^dm^3/n^/g)	2.973	2.413	2.923 × 10^−2^	2.868
	*n_F_*	1.948	1.879	1.126	1.938
	*r* ^2^	0.9440	0.9680	0.9941	0.9422
Redlich−Peterson	*K_RP_* (dm^3^/g)	0.488	0.479	−	0.441
	*a_RP_* (dm^3β^/mg^β^)	6.698 × 10^−3^	1.581 × 10^−2^	−	4.144 × 10^−3^
	*β*	0.883	0.778	−	0.931
	*r* ^2^	0.9993	0.9978	−	0.9949
Temkin	*K_T_* (dm^3^/mg)	3.765 × 10^−2^	3.032 × 10^−2^	4.372 × 10^−3^	3.571 × 10^−2^
	*b_T_* (J g/mol mg)	72.74	72.42	157.1	72.93
	*r* ^2^	0.9839	0.9659	0.8247	0.9883
Dubinin–Radushkevich	*Q_DR(max)_* (mg/g)	286.3	272.9	98.4	284.6
	*K_DR_* (mol^2^/J^2^)	8.110 × 10^−9^	8.369 × 10^−9^	1.616 × 10^−8^	8.209 × 10^−9^
	*E_DR_* (kJ/mol)	7.85	7.73	5.56	7.80
	*r* ^2^	0.9901	0.9971	0.9703	0.9891
Dubinin–Astakhov	*Q_DA(max)_* (mg/g)	210.4	237.9	−	203.0
	*K_DA_* (mol*^nDA^*/J*^nDA^*)	5.462 × 10^−12^	6.442 × 10^−10^	−	2.030 × 10^−12^
	*n_DA_*	2.733	2.257	−	2.834
	*E_DA_* (kJ/mol)	9.34	8.35	−	9.45
	*r* ^2^	0.9988	0.9982	−	0.9996

**Table 4:** Isotherm parameters calculated from linear regression for 18β-glycyrrhetinic acid adsorption onto non-porous silica functionalized with various amine groups.

Adsorption model	Parameter	Adsorbent			
		Aer-AP	Aer-MAP	Aer-DMAP	Aer-AEAP
Langmuir	*Q_L(max)_* (mg/g)	89.3	84.0	−	89.3
	*K_L_* (dm^3^/mg)	2.822 × 10^−3^	2.663 × 10^−3^	−	2.046 × 10^−3^
	*r* ^2^	0.9952	0.9910	−	0.9949
Freundlich	*K_F_* (mg^1−1/n^dm^3/n^/g)	2.179	1.767	1.606 x 10^−2^	1.362
	*n_F_*	2.152	2.058	1.101	1.924
	*r* ^2^	0.9533	0.9730	0.9840	0.9772
Redlich−Peterson	*K_RP_* (dm^3^/g)	0.288	0.284	−	0.221
	*a_RP_* (dm^3β^/mg^β^)	1.250 × 10^−2^	2.345 × 10^−2^	−	1.691 × 10^−2^
	*β*	0.820	0.744	−	0.748
	*r* ^2^	0.9987	0.9982	−	0.9992
Temkin	*K_T_* (dm^3^/mg)	2.942 × 10^−2^	2.294 × 10^−2^	−	1.851 × 10^−2^
	*b_T_* (J g/mol mg)	128.3	124.9	−	116.6
	*r* ^2^	0.9870	0.9731	−	0.9703
Dubinin−Radushkevich	*Q_DR(max)_* (mg/g)	144.9	141.7	65.0	149.8
	*K_DR_* (mol^2^/J^2^)	7.809 × 10^−9^	8.169 × 10^−9^	1.648 × 10^−8^	8.828 × 10^−9^
	*E_DR_* (kJ/mol)	8.00	7.82	5.51	7.53
	*r* ^2^	0.9940	0.9980	0.9376	0.9993
Dubinin−Astakhov	*Q_DA(max)_* (mg/g)	118.2	134.0	−	144.3
	*K_DA_* (mol*^nDA^*/J*^nDA^*)	4.273 × 10^−11^	2.622 × 10^−9^	−	4.375 × 10^−9^
	*n_DA_*	2.525	2.114	−	2.071
	*E_DA_* (kJ/mol)	9.04	8.11	−	7.69
	*r* ^2^	0.9987	0.9982	−	0.9994

**Table 5:** Isotherm parameters calculated from nonlinear fitting analysis for 18β-glycyrrhetinic acid adsorption onto mesoporous silica functionalized with various amine groups.

Adsorption model	Parameter	Adsorbent			
		SBA-15-AP	SBA-15-MAP	SBA-15-DMAP	SBA-15-AEAP
Langmuir	*Q_L(max)_* (mg/g)	183.7	176.7	227.3	182.0
	*K_L_* (dm^3^/mg)	2.357 × 10^−3^	2.033 × 10^−3^	6.453 × 10^−5^	2.278 × 10^−3^
	MPSD	4.66	10.53	11.98	4.03
Freundlich	*K_F_* (mg^1−1/n^dm^3/n^/g)	2.634	2.268	2.921 x 10^−2^	2.479
	*n_F_*	1.918	1.867	1.128	1.895
	MPSD	23.15	18.15	9.36	23.45
Redlich−Peterson	*K_RP_* (dm^3^/g)	0.471	0.468	3.971	0.446
	*a_RP_* (dm^3β^/mg^β^)	5.068 ×10^−3^	1.412 × 10^−2^	135.1	4.516 × 10^−3^
	*β*	0.914	0.790	0.113	0.922
	MPSD	2.72	5.01	9.87	2.11
Temkin	*K_T_* (dm^3^/mg)	5.728 × 10^−2^	5.412 × 10^−2^	−	5.193 × 10^−2^
	*b_T_* (J g/mol mg)	91.96	99.00	−	89.23
	MPSD	22.22	24.26	−	19.16
Dubinin-Radushkevich	*Q_DR(max)_* (mg/g)	282.9	271.5	97.0	282.0
	*K_DR_* (mol^2^/J^2^)	8.107 × 10^−9^	8.358 × 10^−9^	1.654 × 10^−8^	8.234 × 10^−9^
	*E_DR_* (kJ/mol)	7.85	7.73	5.50	7.79
	MPSD	9.74	5.30	20.62	10.16
Dubinin−Astakhov	*Q_DA(max)_* (mg/g)	210.3	237.3	−	202.8
	*K_DA_* (mol*^nDA^*/J*^nDA^*)	5.448 × 10^−12^	6.414 × 10^−10^	−	2.035 × 10^−12^
	*n_DA_*	2.733	2.257	−	2.834
	*E_DA_* (kJ/mol)	9.35	8.37	−	9.44
	MPSD	3.58	4.33	−	1.99

**Table 6:** Isotherm parameters calculated from nonlinear fitting analysis for 18β-glycyrrhetinic acid adsorption onto non-porous silica functionalized with various amine groups.

Adsorption model	Parameter	Adsorbent			
		Aer-AP	Aer-MAP	Aer-DMAP	Aer-AEAP
Langmuir	*Q_L(max)_* (mg/g)	99.6	98.1	−	104.1
	*K_L_* (dm^3^/mg)	2.191 × 10^−3^	1.835 × 10^−3^	−	1.468 × 10^−3^
	MPSD	7.47	11.28	−	10.26
Freundlich	*K_F_* (mg^1−1/n^dm^3/n^/g)	2.011	1.705	1.380 × 10^−2^	1.295
	*n_F_*	2.125	2.052	1.081	1.908
	MPSD	17.73	13.93	15.21	13.33
Redlich−Peterson	*K_RP_* (dm^3^/g)	0.269	0.269	3.910	0.219
	*a_RP_* (dm^3β^/mg^β^)	8.902 × 10^−3^	1.916 × 10^−2^	282.5	1.629 × 10^−2^
	*β*	0.853	0.762	0.075	0.751
	MPSD	3.59	4.65	16.04	2.82
Temkin	*K_T_* (dm^3^/mg)	4.093 × 10^−2^	3.689 × 10^−2^	−	3.117 × 10^−2^
	*b_T_* (J g/mol mg)	148.7	154.9	−	150.1
	MPSD	14.43	17.03	−	18.60
Dubinin–Radushkevich	*Q_DR(max)_* (mg/g)	144.2	141.4	64.0	149.6
	*K_DR_* (mol^2^/J^2^)	7.806 × 10^−9^	8.159 × 10^−9^	1.740 × 10^−8^	8.825 × 10^−9^
	*E_DR_* (kJ/mol)	8.00	7.83	5.36	7.53
	MPSD	6.31	3.70	28.55	2.29
Dubinin–Astakhov	*Q_DA(max)_* (mg/g)	118.2	133.7	-	144.2
	*K_DA_* (mol*^nDA^*/J*^nDA^*)	4.282 × 10^−11^	2.628 × 10^−9^	-	4.363 × 10^−9^
	*n_DA_*	2.525	2.114	-	2.071
	*E_DA_* (kJ/mol)	9.03	8.10	-	7.70
	MPSD	3.03	3.65	-	2.27

**Table 7:** Isotherm model comparison.

Adsorbent	Fitting	
	Linear Regression	Nonlinear Analysis
SBA-15-AP	R-P ≈ L ≈ D-A ≈ D-R > T > F	R-P ≈ D-A ≈ L > D-R > T ≈ F
SBA-15-MAP	D-A ≈ R-P ≈ D-R ≈ L > F ≈ T	D-A ≈ R-P ≈ D-R > L > F > T
SBA-15-DMAP	F > L > D-R > T	F ≈ R-P > L > D-R
SBA-15-AEAP	D-A ≈ L ≈ R-P > D-R ≈ T > F	D-A ≈ R-P ≈ L > D-R > T > F
Aer-AP	D-A > R-P ≈ L ≈ D-R > T > F	D-A ≈ R-P > D-R ≈ L > T > F
Aer-MAP	D-A = R-P ≈ D-R ≈ L > T ≈ F	D-A ≈ D-R ≈ R-P > L > F > T
Aer-DMAP	F > D-R	F ≈ R-P > D-R
Aer-AEAP	D-A ≈ D-R ≈ R-P ≈ L > F ≈ T	D-A ≈ D-R ≈ R-P > L > F > T

Adsorption models: F, Freundlich; D-A, Dubinin-Astakhov; D-R, Dubinin-Radushkevich; L, Langmuir; R-P, Redlich-Peterson; T, Temkin.

## List of Abbreviations and Symbols

AEAPTMS	[3-(2-aminoethylamino)propyl]trimethoxysilane
Aer	Aerosil^®^ (non-porous colloidal silica)
APTMS	(3-aminopropyl)trimethoxysilane
*a_RP_*	constant of Redlich-Peterson isotherm (dm^3β^/mg^β^)
*b_T_*	Temkin constant related to the adsorption heat (J g/mol mg)
BET	Brunauer-Emmett-Teller isotherm
BJH	Barrett-Joyner-Halenda isotherm
*β*	exponential constant of Redlich-Peterson isotherm
18β-GA	18β-glycyrrhetinic acid
*C*_0_	initial adsorbate concentration (mg/dm^3^)
*C_e_*	equilibrium adsorbate concentration (mg/dm^3^)
*C_s_*	solubility (mg/dm^3^)
D-A	Dubinin-Astakhov isotherm
D-R	Dubinin-Radushkevich isotherm
DMAPTMS	(*N,N*-dimethylaminopropyl)trimethoxysilane
DTG	differential thermogravimetry
*E_ads_*	efficiency of adsorption (%)
*E_DA_*	adsorption energy calculated from Dubinin-Astakhov model (J/mol)
*E_DR_*	adsorption energy calculated from Dubinin-Radushkevich model (J/mol)
*ε*	Polanyi potential (J/mol)
F	Freundlich isotherm
IUPAC	International Union of Pure and Applied Chemistry
FT-IR	Fourier-transform infrared spectroscopy
*K_DA_*	constant of Dubinin-Astakhov isotherm related to the adsorption energy (mol^nDA^/J^nDA^)
*K_DR_*	constant of Dubinin-Radushkevich isotherm related to the adsorption energy (mol^2^/J^2^)
*K_F_*	Freundlich constant (mg^1-1/n^dm ^3/n^/g)
*K_L_*	Langmuir constant (dm^3^/mg)
*K_RP_*	constant of Redlich-Peterson isotherm (dm^3^/g)
*K_T_*	Temkin binding constant (dm^3^/mg)
L	Langmuir isotherm
*m*	mass of adsorbent (g)
MAPTMS	[3-(methylamino)propyl]trimethoxysilane
M_18β-GA_	molar weight of 18β-glycyrrhetinic acid molecule (g/mol)
MPSD	Marquardt’s percent standard deviation
*n_DA_*	heterogeneity factor of Dubinin-Astakhov isotherm
*n_F_*	exponential constant of Freundlich equation
*n_FG_*	number of functional groups (mol)
*n_GA_*	number of 18β-glycyrrhetinic acid molecules (mol)
*p/p*_0_	relative pressure
*Q_ads(max)_*	maximum adsorption capacity calculated from given isotherm model (mg/g)
*Q_DA(max)_*	maximum adsorption capacity calculated from Dubinin-Astakhov isotherm (mg/g)
*Q_DR(max)_*	maximum adsorption capacity calculated from Dubinin-Radushkevich equation (mg/g)
*Q_e_*	amount of adsorbate in equilibrium solid state (mg/g)
*Q_FG_*	content of functional groups (mol/g)
*Q_L(max)_*	maximum adsorption capacity calculated from Langmuir equation (mg/g)
*Q_S(max)_*	surface area-normalized maximum adsorption capacity (mg/m^2^)
*R*	gas constant (8.314 J/mol K)
R-P	Redlich-Peterson isotherm
*r*	correlation coefficient
SBA-15	Santa Barbara amorphous (silica)
*S_BET_*	BET surface area (specific surface) (m^2^/g)
STP	standard temperature and pressure
*T*	absolute temperature (K)
T	Temkin isotherm
TEM	transmission electron microscopy
TEOS	tetraethyl orthosilicate
TG	thermogravimetry
V_ads_ N_2_	volume of adsorbed nitrogen (cm^3^ STP/g)
